# Screening of Repellents against Vespid Wasps

**DOI:** 10.3390/insects5010272

**Published:** 2014-03-06

**Authors:** Jean-Luc Boevé, Kris Honraet, Bart Rossel

**Affiliations:** 1 Service Entomology, Royal Belgian Institute of Natural Sciences, Rue Vautier 29, Brussels B-1000, Belgium; 2 Oystershell Laboratories, Booiebos 24, Drongen B-9031, Belgium

**Keywords:** Vespidae, *Vespula*, repellent, volatiles, essential oils, *Mentha*, laboratory testing

## Abstract

Vespid wasps are ecologically beneficial, but they can be a nuisance and dangerous to people due to their tendency to sting. Here, the aim was to screen samples of volatiles (i.e., essential oils and pure chemicals) for their repellency against wasps. The number of wasps (mainly *Vespula vulgaris*) present in a glass box with attractant and 5 µL sample was compared to the number of wasps in a similar box with attractant only. Both boxes were connected to a large glass container harboring 18–35 wasps. Among 66 tested samples, some essential oils from Lamiaceae and Asteraceae, as well as some pure natural compounds such as the monoterpenes (−)-terpinen-4-ol and isopulegol showed a significant repellency against vespids. Our results corroborate the potential of (mixtures of) volatiles in repelling these insects.

## 1. Introduction

Wasps of the family Vespidae (Hymenoptera) can sting in order to attack and in defense, and their painful stings constitute a hazard to humans and other vertebrates [1,2,3,4]. People are exposed to this risk during their professional and recreational outdoor activities, and stings are a real medical concern since some people can die from anaphylactic shock [5,6]. Consequently, methods to monitor and control wasp populations have been developed that are based on fumigation and removal of nests, on trapping in combination with poisoned baits [7,8,9,10,11,12,13,14,15], or on the use of natural enemies such as pathogenic agents [16]. Generally, these methods may be considered as either too destructive, for instance, when the whole wasp nest is destroyed, not effective because wasp colonies can recover [17], or not sufficiently selective since non-pest insects are killed in traps as well [18]. Thus, there is still a need for other controlling methods.

Several Vespidae species live in colonies and are commonly foraging in various natural, agricultural, and urban areas such as forests, orchards, public parks, and private gardens [19]. Notwithstanding their status as a pest, wasps play an important ecological role and they are a major predator of many flies, defoliating caterpillars, *etc*. [20]. They offer perhaps intangible, but substantial “management services” in controlling (other) pest insects but when present as an invasive species, wasps may disturb food webs [21,22,23,24], especially when considering their interactions with honeybees [13,25,26,27,28]. Approximately 20 Vespinae and Polistinae species occur in Europe, but only some of them are considered pests: *Vespula vulgaris* (Linnaeus, 1758), *Vespula germanica* (Fabricius, 1793), *Vespa crabro* Linnaeus, 1758, *Polistes dominulus* (Christ, 1791), and the recently invading *Vespa velutina* Lepeletier, 1836 [29,30]. Ideally, a management program devoted to the control of pestiferous wasps should be adapted to also maintain the biodiversity and the generally beneficial effects of wasps.

The present work was aimed at the screening of wasp repellents to point out the most promising ones, and to this effect we designed a laboratory bioassay. In a recent paper, Zhang *et al*. [31] demonstrated the potential of volatiles as repellents against wasps. These authors identified several essential oils and pure chemicals by combining field trappings and physiological experiments using electroantennogram detection (EAD). We present a laboratory bioassay by which large batches of samples of volatiles can be studied quite conveniently for their potential repellency against wasps.

Volatiles are known to play important roles in the chemical communication of vespid species. They function intra-specifically as alarm [5,32,33,34,35], marking [36] and queen [37] pheromones, while cuticular lipids are implicated in nestmate recognition [38,39] and they include trail pheromones [40,41] and probably sex pheromones [42,43]. Nest-based cues also enable wasps to exploit food resources [44] and volatile acts between species, for instance, when wasps are attracted to plant odors [45,46] by olfactory learning [47]. Moreover, prey pheromones can have a kairomonal effect on wasps, such as a 1:1 mixture of linalool or α-terpineol and (*E*)-2-hexenal that attracts *Vespula maculifrons* [48,49]. However, such wasp communication systems mediated by volatiles are dose-dependent. For instance, venom constituents such as acetals can attract but also alarm or even repel wasps depending on their concentration [50,51].

Here, we designed an *in vitro* bioassay to gradually select samples of volatiles for their repellency on wasp workers. A few active samples were tested after diluting and mixing them in an attempt to infer general trends of the bioactivity of volatiles on wasps. Our findings are discussed from a practical point of view as well as placed in a chemo-ecological context.

## 2. Materials and Methods

### 2.1. Collection of Wasps, and Samples

Vespid wasps were collected in the field using a net during the periods August–September 2011 and July–August 2012 (Table 1) at daytime, generally in the morning. For transport to the laboratory, the wasps were individually placed in plastic vials (diameter 3.5 cm, height 7 cm) and they were provided with a droplet of grenadine syrup. Relatively few wasps could be collected due to bad weather conditions during these two years, forcing us to group individuals from 1–3 species, although each group generally included one major species (Table 1).

**Table 1:** Collection data and numbers of collected wasp species, *Vespula vulgaris* (*V.v.*), *Vespula germanica* (*V.g.*), and *Polistes dominula* (*P.d.*), used in this study. ^a^ Date(s) of collection. ^b^ The four localities mentioned are situated in Belgium. ^c^ Numbers in parentheses correspond to males. ^d^ Unidentifiable specimens.

Group	Date (d/m/y) ^a^	Locality ^b^	Remark	*V.v.*	*V.g.*	*P.d.* ^c^	US ^d^
01	10/08/2011	Vlezenbeek	In and around a garbage container	16	29		
02	16–17/08/2011	Vlezenbeek	As Group 01	6	30		
03	23/08/2011	Vlezenbeek	As Group 01	2	30	1 (+1)	
04	31/08/2011	Ortho	From a nest at the base of a spruce trunk	32			2
05	10–12/09/2011	Vlezenbeek and Uccle	As Group 01, and from wasp traps placed in a garden	6	3	10 (+3)	
06	16–18/07/2012	Vlezenbeek	From a nest at the base of a concrete wall	49			
07	24–26/07/2012	Vlezenbeek	As Group 06	37			
08	31/07–02/08/2012	Vlezenbeek and Uccle	As Group 06, and from wasp traps placed in a garden	26			
09	04–12/08/2012	Vlezenbeek and Uccle	As Group 08	21	1	2	1
10	14/08/2012	Geraardsbergen	From trash cans in a park	26	4		
11	20/08/2012	Geraardsbergen	As Group 10	31	7		
12	27/08/2012	Geraardsbergen	As Group 10	40	2		

Samples of essential oils and pure compounds were obtained from the following companies. Essential oils (possible synonym, plant family): *Juglans regia* (Juglandaceae) (Croda, Nettetal, Germany); Rose Turkish (*Rosa damascena*, Rosaceae) (Danisco, Brugge, Belgium); *Artemisia absinthium* (wormwood), *Artemisia herba-alba* (Asteraceae), *Mentha spicata* (Lamiaceae) and *Zingiber officinalis* (Zingiberaceae) (Essencia Ätherische Öle, Winterthur, Switzerland); *Melaleuca alternifolia* (Myrtaceae) (Kreglinger Europe, Antwerpen, Belgium); *Chamaemelum nobile* (Asteraceae), *Gaultheria procumbens* (Ericaceae), *Juniperus virginiana* (Cupressaceae), *Laurus nobilis* (Lauraceae), *Melaleuca alternifolia* (Myrtaceae), *Mentha arvensis*, *Nepeta cataria*, *Origanum majorana* (Lamiaceae), *Pinus sylvestris* (Pinaceae) and *Valeriana officinalis* (Valerianaceae) (Pranarôm International, Ghislenghien, Belgium); Hippophae (sea buckthorn, Elaeagnaceae) (Safic-Alcan, Londerzeel, Belgium); *Lavendula angustifolia* (Lamiaceae) (Sensient Essential Oils, Bremen, Germany); *Caryophyllus aromaticus* (Myrtaceae), *Cymbopogon nardus* (Poaceae) and *Helichrysum italicum* (Asteraceae) (Sjankara, Tielt, Belgium); and Rosae aetheroleum (Rosaceae) (Synaco, Knokke-Heist, Belgium).

Mixtures not as essential oils: pyrethrum extract (*Chrysanthemum*, Asteraceae, 0.05% w/w in isododecane) (Kenya Pyrethrum Information Centre, Kuchl, Austria); pitch-oil (Pohjolan Terva, Kursu, Finland); Carnation^®^ (Sonnneborn, Amsterdam, The Netherlands); and sunflower oil (*Helianthus annuus*, Asteraceae) (Vandemoortele, Izegem, Belgium).

Pure chemicals (synonym, purity as far as known, possible solid phase): 7(*Z*)-pentacosene (Bio-Connect, Huissen, The Netherlands); isopropyl alcohol (Conforma, Destelbergen, Belgium); Frescolat^®^ MGA and Frescolat^®^ ML (crystals) (Cosnaderm, Amsterdam, The Netherlands); 3-methylpentacosane (solid) and 7-ethyl-2-methyl-1,6-dioxaspiro(4,5)decane (Ecosynth, Oostende, Belgium); lilial and alpha-irone (Essencia Ätherische Öle, Winterthur, Switzerland); *N*-(3-methylbutyl)acetamide (Frinton Laboratories, Hainesport, NJ, USA); menthyl PCA (Questice^®^, 25% in ethanol) (GOVA, Antwerpen, Belgium); isododecane and isoeicosane (IMCD, Wormermeer, The Netherlands); diisopropyl adipate (Ceraphyl^®^ 230) (Keyser & Mackay, Bruxelles, Belgium); propylene glycol (Mosselman, Ghlin, Belgium); ethyllactate (Purac, Gorinchem, The Netherlands); saltidin (picaridin, icaridin) (Saltigo, Einsiedeln, Switzerland); (−)-terpinen-4-ol, 2-heptanone, 2-nonanone (99%), benzaldehyde, camphor (96%, solid), carvone, eucalyptol (99%), eugenol (99%), heptyl butyrate (98%), isopulegol, linalool, menthol (solid), menthone, methyl anthranilate, methyl salicylate, myrcene (85%), *N*-ethyl-2-isopropyl-5-methylcyclohexane, octanoic acid, oleic acid, thymol (99.5%), (+)-*cis*-*p*-menthane-3,8-diol, and triethyl citrate (98%) (Sigma-Aldrich, Diegem, Belgium); DEET (Vertellus, Antwerpen, Belgium); IR3535 (VWR International, Haasrode, Belgium).

### 2.2. Experimental Setup

**Figure 1:**
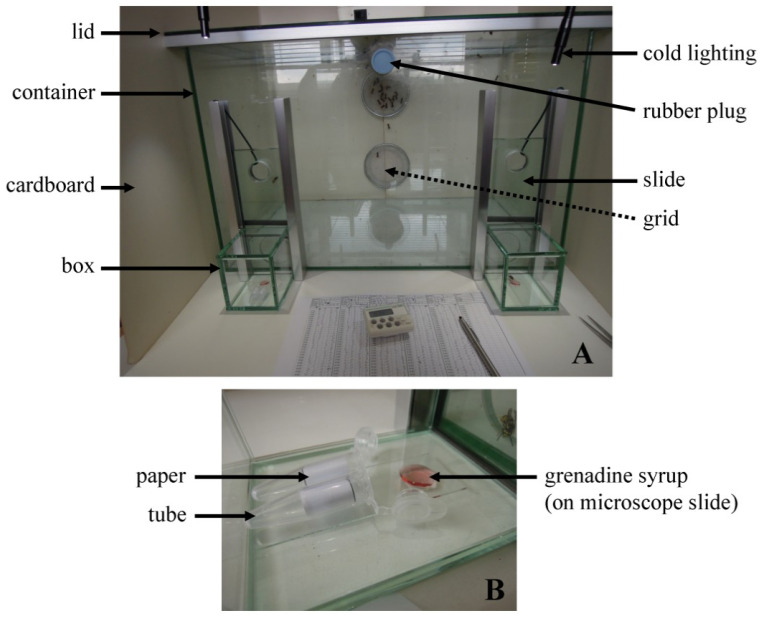
Annotated photography of the bioassay set-up. (**A**) Overviewing picture showing the glass container and, among others, two boxes on the left and right sides; note that the glass reflects the light coming from a window. (**B**) One of the two boxes ready for testing a sample on the wasps; this picture represents the left box, thus with the container on the right side of the picture.

A glass container (width 60 cm, depth 40 cm, height 40 cm) was composed of fixed walls and floor, and a removable lid (Figure 1A). It had five holes. Three holes were located on the front wall: two low, left and right, and one small high and centered. The back wall had two larger holes which were covered by a stainless steel grid. A removable glass box (8 × 8 × 10 cm) could be attached to the container in front of each of the two lower front holes with the open side facing the container (Figure 1B). A removable glass plate (width 10 cm, height 15 cm) could then be used as a sliding door between the container and the box. A cold light source was directed via two gooseneck arms from above on each one of these boxes. The three walls (left, right and behind) and the top of the container were protected from light with white cardboard covers so that the only light allowed came from the front of the container (Figure 1A). A ventilator was used by removing the cardboard from the back wall and directing the airflow into the container through the lower grid covered hole. Note that by doing so, the airflow venting from the higher grid-covered hole could clearly be detected.

### 2.3. Bioassay

At least 18 wasps from one group were transferred into the glass container via the central, front hole that was otherwise closed with a rubber plug (Figure 1A). The number of wasps in the container varied during the period of testing (i.e., maximum one week; see later). More wasps could be added, sometimes to replace dead or moribund ones. The latter, if possible, were taken out of the container and kept in ethanol for later identification (Table 1). Wasps were also added to compensate an overall activity decrease in the container. During the night the wasps were provided with grenadine syrup and water.

In order to test the samples, a 1.5 × 3.0 cm piece of paper (Dolphin Premium, 80 g m^−2^; Dolphin Papers, Franklin, IN, USA) was rolled and placed into a plastic Eppendorf tube (vol. 1.5 mL). For each experiment, two plastic tubes were weighed together (i.e., tare weight). Then, in each tube 2.5 µL of the sample were deposited on the paper, preliminary tests showing that in this way the evaporation (of 5 µL) was enhanced. The two tubes were then immediately closed and weighed again together (start weight). In each glass box, a microscope glass slide was placed, and 200 µL of grenadine syrup (i.e., attractant) was deposited on one end of the slide. Then, the two tubes were opened and placed in one of the two glass boxes, on the slide (see positioning in Figure 1B). The box with the tubes was fixed at random on the left or right side of the container. Both boxes were attached to the container and the sample was allowed to evaporate inside the box for 2 min. Afterwards, the glass plates were drawn and held upwards (see Figure 1A) so that the wasps could get into the boxes. The time point of opening was set as t = 0. The number of wasps in each box was then counted and recorded every 10 s, from t = 10 s to t = 5 min (i.e., 30 matched pairs of counts; first run). Prior to closing off the boxes with the removable slides, we made sure all the wasps had withdrawn back into the main container. This could take a few minutes and wasps could be coaxed back by using a light source directed from the backside of the container. Then, the boxes were removed from the container, the two tubes taken out, closed and weighed together (intermediate weight). The container was ventilated for at least 1 min and the two boxes were ventilated for at least 5 min. On rare occasions, if more than 50% of the sample had already evaporated, an additional 2 × 2.5 µL of sample were added to the paper in the tubes that were then weighed again (after this extra load). The tubes were placed again in one of the two boxes, the right box if the sample was first tested on the left side, and *vice versa*. The same test was then performed in a second run after which the two tubes were weighed for a last time (final weight). The temperature inside the container was recorded. After an “experiment” (i.e., two successive runs), the two tubes were discarded, and the microscope slides were cleaned. The succession of samples used in the experiments was determined at random. Wasps were used during 2–7 days, after which they were killed, kept in ethanol for identification (see Table 1), and replaced by new ones, collected in the field.

### 2.4. Screening of Volatiles

The samples were mainly chosen in an exploratory way by including common essential oils and a series of pure chemicals. Some were chosen because they reportedly modulate behavioral processes of vespids (e.g., [34,35,39]), others because they are mentioned in the EU Biocidal Product Directive, or because they have a known insecticidal activity.

Each sample was used in at least two experiments. It was then selected for retesting if it showed a significant repellent activity with the Sign test in both experiments (see later).

### 2.5. Dilutions and Mixtures of Selected Samples

From the screening process, four of the most active samples were chosen for further analysis. The samples were diluted in propylene glycol (10%, 17.8%, 31.5%, and 56.2%) and the repellency of the dilutions was determined with the bioassay. The repellency of the six possible combinations of two by two mixtures (vol. 1:1) of the same samples was determined as well. The experiments were performed in triplicate (i.e., six runs per dilution or mixture).

### 2.6. Statistics and Calculations

For each experiment, the double (from two runs) series of 30 matched pairs of wasp numbers were averaged per min and rounded to the nearest integer, and these 10 pairs of values were used in the non-parametric Sign test [52]. The Sign test offered the advantage to be applicable per experiment, thus allowing a daily statistical check of which samples were to be tested further.

However, drawbacks of the Sign test in this setup are the temporal auto-correlation of the data since successive numbers of wasps could (partly) correspond to the same wasp individuals and since not (all) the same wasps were participating in the two runs. Therefore, the overall screening dataset was analyzed with R version 2.15.3 and from which the following values were extracted. The performance of the experiment itself was determined at the control side by the total time without wasps (TTWW) and the maximum number of wasps (MNW). Note that a low TTWW and high MNW are desirable since this reflects frequent wasp visits and a high number of wasps visiting, meaning high wasp participation. A repellent potency was also computed based on a weighted score taking into account the 30 time points for each of the (test and control) sides, and assigning more weight to the absence of wasps (as for the TTWW). This score was set equal to: (N_0_ × 4) + (N_1_) + (N_2_ × 0.5) + (N_3_ × 0.25) + (N_>3_ × 0.1), where N_y_ is the number of time points with y wasps. The repellent potency was the difference between the scores of the test and control side. Since the expected minimum and maximum scores were 3 and 120 the repellent potency could vary from −117 to 117, the latter denoting the highest possible repellent potency.

From each experiment the evaporation rate (in %) of the sample was calculated with the formula: [(final weight − start weight)/(final weight − tare weight)] × 100. The rate was adjusted in those cases where the samples needed to be replenished.

## 3. Results

### 3.1. Repellency of Screened Samples

**Figure 2:**
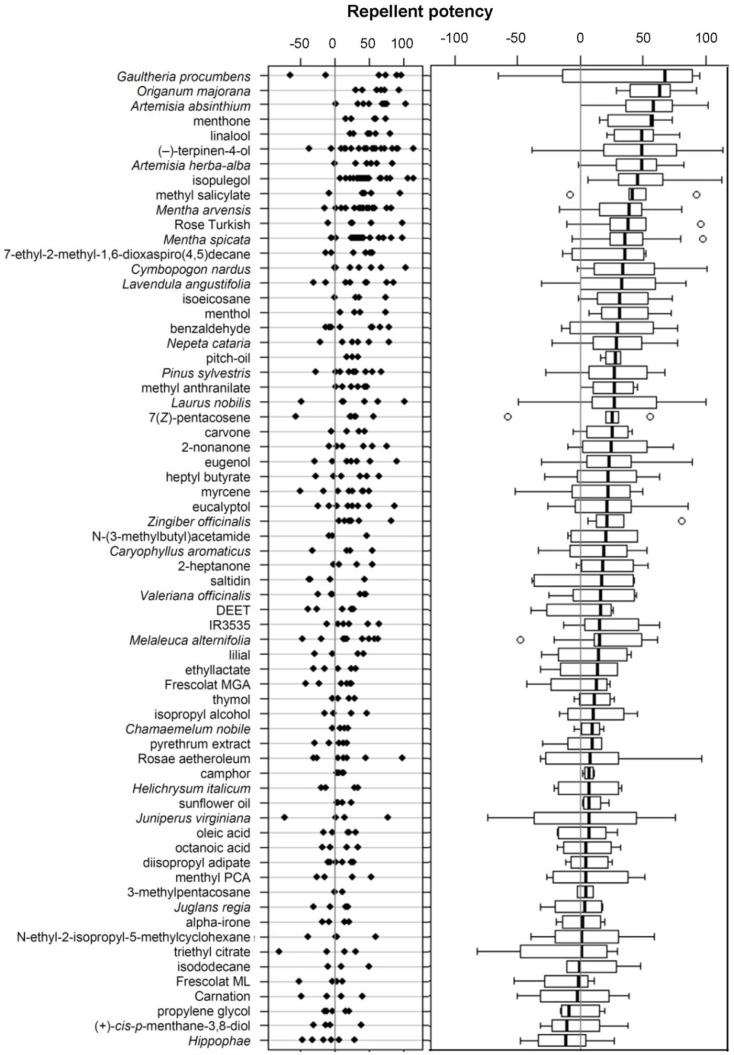
Samples screened for their repellent potency against wasps. Dot plot (left diagram) and box-and-whisker plot (right diagram) of the repellent potencies. Samples are ordered according to a decreasing median of the repellent potency. Each dot in the dot plot represents the repellent potency calculated from one run.

From 208 screening experiments performed in 2011 and 2012 on a total of 66 samples, only a minority were statistically significant in the Sign test. Hence, most samples were tested not more than three times. The repellent potency of all tested samples (Figure 2) revealed that the 10 most (consistent) repellent samples were the essential oils of *G. procumbens*, *O. majorana*, *Artemisia* spp., and *M. arvensis*, as well as the chemicals menthone, linalool, (−)-terpinen-4-ol, isopulegol, and methyl salicylate. Strikingly, the “repellents” of the PT19 class of the annex II of the BPD, such as DEET, IR3535, saltidin, and (+)-*cis*-*p*-menthan-3,8-diol, showed (almost) no repellent potency against wasps (Figure 2).

### 3.2. Repellency and Evaporation Rates

**Figure 3:**
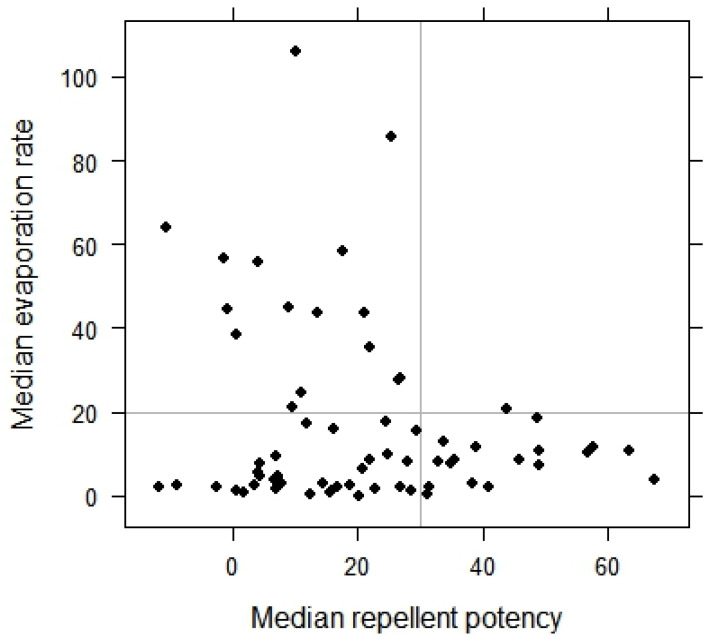
Distribution of the samples as a function of their repellent potency against wasps and their evaporation rate.

During the experiments, the temperature in the container ranged from 22–32 °C and the evaporation rate of the samples ranged from 0–96%. In eight experiments, the sample was reapplied to the paper in the tubes before performing the second run of the experiment, and in these cases, 49%–92% of the sample evaporated during the two runs of the experiment. Considering all samples, a high repellent potency (>30) was associated with a low evaporation rate (≤20%) (Figure 3).

### 3.3. Repellency of Dilutions and Mixtures

The most consistent repellent samples tested during 2011 were two mint oils, *M. spicata* and *M. arvensis*, and the two chemicals isopulegol and (−)-terpinen-4-ol. These four samples were chosen during 2012 to be tested as dilutions and mixtures. The samples were generally less active once diluted, but a dose-dependent decrease in activity was clear only for *M. spicata* (Table 2). Further, mixing these four samples two by two in all six possible combinations never resulted in an increase of repellent potency (Table 3).

**Table 2:** Repellent potency of four samples as a function of their concentration. Values given as repellent potency (mean ± SD). Values in square brackets are the number of runs performed. ^b^ Log values of the sample diluted in propylene glycol.

Essential oils and pure chemicals	Concentration ^b^				
	0.00	−0.25	−0.50	−0.75	−1.00
*Mentha spicata*	38.3 ± 27.3	28.3 ± 25.0	24.1 ± 17.7	21.8 ± 27.4	−7.6 ± 19.1
	[18]	[6]	[6]	[6]	[6]
*Mentha arvensis*	36.3 ± 24.9	8.7 ± 14.1	0.3 ± 9.6	1.7 ± 13.1	2.3 ± 8.5
	[18]	[8]	[6]	[6]	[6]
(−)-terpinen-4-ol	47.4 ± 37.7	18.0 ± 19.3	28.4 ± 34.2	7.0 ± 13.2	8.5 ± 38.0
	[20]	[6]	[8]	[6]	[6]
isopulegol	49.9 ± 28.9	10.0 ± 36.1	15.5 ± 9.3	25.2 ± 14.4	−5.3 ± 48.2
	[18]	[6]	[6]	[6]	[6]

**Table 3:** Repellent potency of four samples tested by mixing them two by two. All mixtures in volume 1:1. Values are given as repellent potency (mean ± SD) above the names of the essential oils and pure chemicals, whereas values in square brackets and below these names are the number of runs performed.

*Mentha spicata*	20.0 ± 34.1	15.7 ± 17.8	16.4 ± 21.9
[6]	*Mentha arvensis*	8.2 ± 34.0	36.4 ± 23.4
[6]	[6]	(−)-terpinen-4-ol	−2.3 ± 7.0
[6]	[6]	[6]	isopulegol

### 3.4. Bioassay Assessment

Besides the repellence itself of the samples, we detected four factors which potentially influenced the results. First, not all wasps participated equally in the experiment, with wasps of groups 3, 4 and 10–12 participating the most (Table 4). Second, each group was characterized by a predominant wasp species (see Table 1): *V. germanica* for groups 1–3, *V. vulgaris* for groups 4 and 6–12, and *P. dominula* for group 5. The lowest TTWW and highest MNW were obtained with *V. vulgaris* as compared to the two other species (Table 5). Third, a side preference was often observed, that is wasps tended to frequent the right box slightly more than the left box (TTWW = 26.2 ± 44.0 s for right against 42.8 ± 52.3 s for left). This may lead to overestimating the repellent potency of samples on the left side. Fourth, after a couple of days, wasps tended to become immobile and stayed in a cluster on the grids (as shown in Figure 1A), or at the underside of the container’s glass lid.

**Table 4:** Statistical values as a function of the 12 groups of wasps. ^a^ Total time without wasps (sec). ^b^ Maximum number of wasps.

Group	Runs (*N*)	Mean ± SD		Median		Min–Max	
		TTWW ^a^	MNW ^b^	TTWW ^a^	MNW ^b^	TTWW ^a^	MNW ^b^
01	56	52.1 ± 56.0	4.2 ± 1.8	30	4	0–210	1–9
02	54	65.4 ± 70.6	4.5 ± 2.4	45	4	0–250	2–12
03	32	21.3 ± 38.8	6.0 ± 2.4	0	6	0–130	2–12
04	50	23.6 ± 36.4	5.5 ± 1.9	10	5	0–200	2–9
05	16	45.6 ± 45.2	3.6 ± 1.3	30	4	10–190	2–7
06	20	37.0 ± 49.2	5.1 ± 2.3	20	4	0–170	1–9
07	46	38.7 ± 43.0	4.4 ± 1.7	20	4	0–160	2–9
08	56	44.1 ± 70.0	4.8 ± 1.9	15	5	0–300	0–10
09	22	59.1 ± 58.1	3.4 ± 1.3	45	3	0–220	1–6
10	64	15.2 ± 17.8	6.4 ± 1.8	10	6	0–70	3–10
11	76	17.0 ± 22.2	6.3 ± 2.0	10	6	0–110	3–13
12	66	25.0 ± 34.4	6.1 ± 2.0	10	6	0–150	2–12

**Table 5:** Statistical values as a function of the predominant wasp species in the groups. ^a^ Total time without wasps (sec). ^b^ Maximum number of wasps.

Wasp species	Runs (*N*)	Mean ± SD		Median		Min–Max	
		TTWW ^a^	MNW ^b^	TTWW ^a^	MNW ^b^	TTWW ^a^	MNW ^b^
*Vespula vulgaris*	400	28.5 ± 42.8	5.5 ± 2.1	10	5	0–300	0–13
*Vespula germanica*	142	50.2 ± 60.9	4.7 ± 2.3	30	4	0–250	1–12
*Polistes dominula*	16	45.6 ± 45.2	3.6 ± 1.3	30	3.5	10–190	2–7

## 4. Discussion

The present study evidences that both essential oils and pure chemicals can act as repellents, but a majority of the 66 tested samples were not repellent in each run. This may be partly explained by the fact that the amount of 5 (= 2 × 2.5) µL sample was rather low. A 1:1 mixture of (−)-terpinen-4-ol and isopulegol was tested in three experiments by using 10 (= 2 × 5) µL and it showed each time a significant repellency (i.e., *p* ≤ 0.004 three times; Sign test, two-tailed). From 5 µL, however, the repellent potency decreased rapidly upon dilution of such chemicals. Thus, the 5 µL used in the screening assay is a quite low amount. Since samples were also less active once mixed, it seems that they have neither a synergistic, nor an additive repellent activity.

The four aforementioned factors that may have influenced our experimental results were also counteracted by the bioassay setup itself, as follows: the succession of testing samples was randomized within and among the groups of wasps; test and control sides were always switched between the left and right boxes (i.e., the first and second run within each experiment); when the wasp activity decreased over time, fresh wasps were released in the container. Several negative controls were performed by testing unloaded instead of loaded vials, and they always led to non-significant results (data not shown). Further, different vespid species were often tested simultaneously, but they are known to react similarly towards repellents [31], and aggressive interactions were rarely observed in the container and boxes.

Some essential oils and their respective major constituents both exhibited a high repellent potency. This was the case, for instance, for *G. procumbens* and methyl salicylate, or *M. arvensis* and menthol ([53]; Figure 2). The latter compound is a characteristic constituent of the essential oil of peppermint (*Mentha* spp.), and five of its analogues were tested. Surprisingly, these (six) substances were strongly dissimilar in their repellency against wasps. Only menthone was in the same range of activity as menthol (Figure 2), hinting that their common part, 5-methyl-2-isopropylhexane, contributes importantly to this bioactivity.

Volatiles can have opposite effects on vespids depending on their concentration and on the context in which they are emitted and perceived. For instance, spiroacetals are constituents of the venom and alarm conspecifics, while they have been used as wasp attractants [35,51]. Wasps are attracted to green-leaf volatiles [48,49] and it is assumed that such compounds indirectly indicate the feeding activity of phytophagous insects that constitute potential prey. They are also attracted to plant odors indicating a source of carbohydrates [47]. For instance, leaves of catmint, *N. cataria*, can become highly attractive to wasps through a sugar rewarding [47], while the essential oil of this plant was found to be a moderate or weak repellent (Figure 2), and its major compound, nepetalactone, as a strong or moderate repellent [31]. Such dissimilar behavioral responses are not inconsistent with EAD responses of wasps (see [31]), because chemicals can trigger an antennal reaction, regardless of being a repellent, or attractant. The general physiological state of the wasps should also be considered. Wasps tested in the present study were provided with grenadine syrup, which progressively decreased their satiation level (especially if compared to wasps in nature). We did not consider learning or habituation processes towards neither grenadine syrup that is a fruit blend, nor the chemicals/samples themselves, and both aspects would require further testing.

## 5. Conclusions

Converting experimental results into an applied solution to combat the hazard of vespids remains a challenging task. On the one hand, the use of volatiles should be promoted, regarding, generally, the ecological importance of vespids in limiting the impact of other pest insects. The aforementioned remarks on the chemical ecology of wasps lead, however, to the general conclusion that numerous aspects will affect the final effectiveness of a commercial product, depending also on the application strategy. One option is the “repel and attract (i.e., push-pull) strategy” where wasps are kept away from people as well as attracted to poisoned traps [54]. However, just a “repel strategy” would not only be ecologically more relevant, but also sufficient when people spend time outdoors. On the other hand, the application of strongly scented essential oils may be offensive to repellent users so that it is important to carefully select the least offensive repellents. The present study reveals essential oils and pure chemicals as effective repellents, and some of these may be especially promising in that their level of perception by humans can be low. One should also put in balance agricultural and/or industrial processes, economic costs, and the environmental impact for each type of chemicals, to optimize the formulation of volatiles so that humans can protect themselves, in a sustainable way, against the hazard of pestiferous wasps.
